# Identification of the Promoter Antisense Transcript Enhancing the Transcription of the Equine Herpesvirus-1 Immediate-Early Gene

**DOI:** 10.3390/v16081195

**Published:** 2024-07-25

**Authors:** Mayuko Maeda, Miou Abe, Keisuke Aoshima, Atsushi Kobayashi, Hideto Fukushi, Takashi Kimura

**Affiliations:** 1 Laboratory of Comparative Pathology, Faculty of Veterinary Medicine, Hokkaido University, Sapporo 060-0818, Japan; mykmd.schnee@gmail.com (M.M.); mioumurashita@gmail.com (M.A.); k-aoshima@vetmed.hokudai.ac.jp (K.A.); kobayashi@nagasaki-u.ac.jp (A.K.); 2 Laboratory of Veterinary Microbiology, Faculty of Applied Biological Sciences, Gifu University, Gifu 501-1193, Japan; fukushi.hideto.r9@f.gifu-u.ac.jp

**Keywords:** equine herpesvirus-1, gene expression, non-coding RNA

## Abstract

Equine herpesvirus-1 (EHV-1) causes respiratory diseases, abortion, and encephalomyelitis in horses. The EHV-1 immediate-early (IE) protein, essential for viral replication, is transactivated by the binding of a multiprotein complex including the open reading frame 12 (ORF12) and some host factors to the IE promoter region. Promoter-associated non-coding RNAs (pancRNAs), which are transcribed from bidirectional promoters, regulate the transcription of neighboring genes in mammals and pathogens. In this study, we identified a novel pancRNA transcribed from across the areas of the 5′-untranslated region and a promoter of EHV-1 IE and named it IE pancRNA. IE pancRNA and mRNA were simultaneously expressed in EHV-1-infected RN33B-A68B2M cells. This pancRNA was also transcribed in RK13 and E. Derm cells, which are highly susceptible to EHV-1 infection. Furthermore, IE pancRNA upregulated *IE* gene expression in the presence of ORF12, and stable expression of IE pancRNA increased the number of EHV-1-infected RN33B-A68B2M cells. These results suggest that IE pancRNAs facilitate EHV-1 proliferation by promoting *IE* gene expression.

## 1. Introduction

Equine herpesvirus-1 (EHV-1), an alphaherpesvirus belonging to the *Herpesviridae* family, is distributed worldwide and causes respiratory diseases, abortion, and encephalomyelitis (equine herpesvirus myeloencephalopathy) in horses. EHV-1 is a serious problem in the equine industry and causes significant economic losses. EHV-1 is transmitted via the respiratory pathway. This virus causes latent infections in horses, leading to periodic reactivation as reservoirs of infection for other susceptible horses. Latent infections have been reported in the lymphoid and neural tissues [1,2].

During lytic infection, EHV-1 genes are coordinately regulated in the following phases: immediate-early (IE), early, and late phases [3,4]. The *IE* gene, which maps within each of the two inverted repeat sequences, is the first gene to be expressed in all EHV-1 coding genes [4,5], from which 6.0-kb transcripts are synthesized [4]. EHV-1 mutants, lacking the *IE* gene, cannot replicate in cultured cells, and no viral protein synthesis is detected in such cells, suggesting that *IE* is essential for EHV-1 replication [6]. Transcription of EHV-1 *IE* gene is activated by the open reading frame 12 (ORF12) protein, a functional homolog of α-trans-inducing factor (TIF) gene in herpes simplex virus (HSV) type 1 [7,8]. Similar to α-TIF, ORF12 forms a multiprotein complex with host cellular factors, including OCT-1 and HCF, on DNA-binding sites to interact with the IE promoter and enhance its transcription [9]. α-TIF plays important roles in reactivating latent HSV and causing infections [10].

Viral non-coding RNAs (ncRNAs) are vital for viral infections [11,12]. Several types of viral ncRNAs have been reported to affect viral replication [13]. For example, the HSV-1 latency-associated transcript, which is processed into several microRNAs (miRNAs) and is the only viral gene expressed during latent infection in neurons, inhibits apoptosis and reduces viral protein expression to maintain latency [14,15]. Kaposi’s sarcoma-associated herpes virus polyadenylated nuclear RNA interacts with demethylases and physically binds to promoter regions to mediate viral gene expression [16]. Furthermore, miR-U86, an miRNA encoded by human herpes virus 6A, inhibits viral lytic replication by regulating the *IE* gene [17]. However, the specific roles of ncRNAs in EHV-1 infection remain unclear.

Promoter-associated ncRNAs (pancRNAs) are ncRNAs transcribed within a few hundred bases of the transcription start sites (TSSs) of protein-coding or non-coding genes [18,19]. pancRNAs affect the promoter region and play an important role in the epigenetic regulation of neighboring protein-coding gene expression [18]. For example, antisense pancRNA expression from the promoter regions of neurofilament light chain (*Nefl*) and *Il17d* induces DNA demethylation in the promoter region to promote transcription [20,21]. However, the roles of pancRNAs in viral infections remain poorly understood.

In this study, we aimed to identify novel pancRNAs from the IE promoter region of EHV-1 and analyze their roles in *IE* gene expression.

## 2. Materials and Methods

### 2.1. Cells and Viruses

Rn33B-A68B2M cells previously established and stored in our laboratory [22] were used. RK13 and E. Derm cells were obtained from the American Type Culture Collection (Manassas, VA, USA). Rn33B-A68B2M cells were cultured in a mixture of Dulbecco’s modified Eagle’s medium (DMEM; Nissui, Tokyo, Japan) and Ham’s F-12 (Nissui) in a 1:1 ratio supplemented with 10% fetal bovine serum (FBS; Biowest, Logan, UT, USA) at 32 °C with 5% CO_2_. Differentiation of Rn33B-A68B2M cells was performed as described previously [22]. Briefly, cells were cultured using high-glucose DMEM (FUJIFILM Wako Pure Chemical Corporation, Osaka, Japan) and Ham’s F12 in a 1:1 ratio containing 1% bovine serum albumin (FUJIFILM Wako) and N_2_ supplemented with transferrin (Holo; FUJIFILM Wako) at 37 °C with 5% CO_2_ for two days. RK13 cells were cultured in the Eagle’s minimum essential medium (Nissui) containing 10% FBS at 37 °C with 5% CO_2_. E. Derm cells were cultured in DMEM containing 10% FBS and MEM non-essential amino acid solution (FUJIFILM Wako) at 37 °C with 5% CO_2_. EHV-1 strain Ab4 [23] and EHV-1 mutant Ab4-green fluorescent protein (GFP) containing a GFP expression cassette between open reading frames (ORFs) 62 and 63 [24] were used. Stock viruses were propagated in RK13 cells and titrated using a plaque formation assay in RK13 cells.

### 2.2. Primers and Synthetic dsDNA

All primers used for polymerase chain reaction (PCR) and rapid amplification of cDNA ends (RACE) were synthesized by Eurofins Genomics (Tokyo, Japan). All primers used in this study are listed in Table 1. Long synthetic dsDNA was obtained from GenScript Biotech Corporation (Tokyo, Japan).

### 2.3. Reverse Transcription-Quantitative PCR (RT-qPCR)

Undifferentiated and differentiated Rn33B-A68B2M cells were cultured at 32 and 37 °C, respectively. Two days after seeding, the cells were infected with EHV-1 Ab4 at a multiplicity of infection (MOI) of 5. After incubation at 37 °C for 1 h, cells were washed with phosphate-buffered saline (PBS) three times and cultured with a growth medium. At 24 h post-infection (p.i.), the cells were collected, and total RNA was extracted using NucleoSpin RNA (Takara, Shiga, Japan) according to the manufacturer’s instructions. Complementary DNA (cDNA) was synthesized using PrimeScript II first-strand cDNA Synthesis Kit (Takara) from 1 μg of total RNA of samples. cDNA (1 μL) was used at a final reaction volume of 5 μL, and the experiments were performed in triplicate using KAPA SYBR FAST qPCR Master Mix (2×) ABI Prism (KAPA Biosystems, Wilmington, MA, USA) and primers for EHV-1 IE promoter_F and EHV-1 IE promoter_R (Table 1) in a Step One Real-Time PCR system (Thermo Fisher Scientific, Waltham, MA, USA). The real-time PCR program was as follows: 95 °C for 3 min and 40 cycles of 95 °C for 3 s (s) and 60 °C for 20 s. Rat *β-actin* was used as an internal control and was amplified using the primers Rat_*β*-actin_F and Rat_*β*-actin_R (Table 1). The *C_t_* values of the target genes were normalized using those of internal controls. Subsequently, we compared the expression levels of genes using the 2^−ΔΔ*Ct*^ values.

### 2.4. RACE

5′- and 3′- RACE for ncRNA was performed using a SMARTer RACE 5′/3′ Kit (Takara) according to the manufacturer’s instructions. Briefly, undifferentiated Rn33B-A68B2M cells were infected with EHV-1 Ab4 at an MOI of 5. At 24 p.i., cells were collected, and total RNA was extracted using NucleoSpin^®^ RNA according to the manufacturer’s instructions.

For 5′ RACE, The 5′-first strand cDNA synthesis was performed with random primers. The PCR reaction was performed with the Universal Primers Mix provided in the kit and 5′ RACE GSP of 5′ RACE Antisense GSP, 5′ RACE Sense GSP-1 or 5′ RACE Sense GSP-2 (Table 1).

For 3′RACE, poly(A) tailing was performed by using Poly(A) Tailing of RNA using E.coli Poly(A) Polymerase (New England Biolabs Japan, Tokyo, Japan) because ncRNA possibly lacks a polyadenylated tail. Reverse transcription was performed with the 3′-CDS primer A provided in the kit. The PCR reaction was performed with the Universal Primers Mix and 3′ RACE GSP of 3′ RACE Antisense GSP, 3′ RACE Sense GSP-1, 3′ RACE Sense GSP-2, and 3′ RACE Sense GSP-3 (Table 1). All the amplified products were cloned into a pRACE vector and sequenced.

### 2.5. DNA Cloning and Preparation of RNA Probes

The cDNA fragment of ncRNA (designated as IE promoter-associated ncRNA; IE pancRNA in the Results section) was amplified via PCR using synthetic dsDNA spanning the entire sequence of the ncRNA transcripts as a template. cDNA fragments of EHV-1 IE and rat *β-actin* were synthesized using reverse transcription PCR. For the EHV-1 *IE* gene, cDNA was synthesized from total RNA of EHV-1-infected RK13 cells. For the rat *β-actin* gene, cDNA was synthesized from the total RNA of undifferentiated Rn33B-A68B2M. The primer pairs used for amplification were as follows: for the probe complementary to the 5′ side of IE pancRNA, 5′-GGCGAATTCCTCTTGGCACTCCTTCTTCG-3′ and 5′-ACTCAAGCTTCTTCGAGGTAAGTATCCCCAC-3′; for the probe complementary to the 3′ side of IE pancRNA, 5′-GGCGAATTCCTTTAATGAGATTCAACCGGG-3′ and 5′-ACTCAAGCTTCCGCTCATATGCATAAAGACG-3′; for IE mRNA, 5′-TCGCCGCGATGCTGAAGATG-3′ and 5′-TTCGTCGCTGTCGCTGTCGT-3′; for *β-actin*, 5′-ACTCAAGCTTAGGCCAACCGTGAAAAGATG-3′ and 5′-GGCGAATTCAGTCTAGGGCAACATAGCAC-3′. The PCR products of IE pancRNA and rat *β-actin* were cloned into the EcoRI-HindIII site of the pGEM-3Z vector (Promega, Madison, WI, USA). The PCR products of *IE* mRNA were cloned into the pGEM-T vector (Promega).

Antisense RNA probes were prepared using the digoxigenin (DIG) RNA-labeling kit (SP6/T7) (Roche, Basel, Switzerland). To obtain templates for RNA transcription, the plasmid DNA containing the PCR product was linearized with the restriction enzyme HindIII or NotI. Each linearized DNA fragment was labeled using the T7 transcription runoff method by incorporating DIG-11-UTP into single-stranded specific RNA probes. The labeled probes generated from 3 μg of the plasmid DNA were precipitated with ethanol and then dissolved in 50 μL of RNase-free water. The RNA probes were stored at −80 °C.

### 2.6. Northern Hybridization

Total RNA was extracted using TriPure Isolation Reagent (Roche) from EHV-1-infected or -uninfected Rn33B-A68B2M, RK13, and E. Derm cells according to the manufacturer’s instructions. Hybridization was performed using the method described by Shifman and Stein [25] with slight modifications. Briefly, 1 μg of RNA samples were electrophoresed through a 1.5% agarose-2.2M formaldehyde gel and were transferred to nylon membranes (positively charged; Roche). The RNAs were fixed to the membrane using an XL-1000 UV crosslinker (Spectronics Corporation, Lincoln, NB, USA) with subsequent baking at 80 °C for 2 h. Membranes were prehybridized in 0.25 M Na_2_HPO_4_ (pH 7.2), sodium dodecyl sulfate (SDS) 10%, 1 mM EDTA, and blocking reagent 2% at 68 °C for 3 h. Hybridization was conducted in the same buffer containing 6–8 ng/mL of the DIG-labeled cRNA probe at 68 °C for 15 h. After hybridization, membranes were washed three times for 20 min each in 25 mM Na_2_HPO_4_ (pH 7.2), SDS 1%, and 1 mM EDTA at 68 °C. Hybridization signals were visualized using an alkaline phosphatase-conjugated anti-DIG antibody and disodium 3-(4-methoxyspiro{1,2-dioxetane-3,2′-(5′-chloro)tricyclo [3.3.1.1^3,7^]decan}-4-yl) phenyl phosphate (CSPD) chemiluminescent substrate (Roche).

### 2.7. Expression Vector and Reporter Plasmid Construction

The pcDNA3.1 (+) vector encoding full-length IE pancRNA was generated using GenScript. This plasmid was designated pcDNA-IE pancRNA. EHV-1 ORF12 gene was amplified from EHV-1 HH1 strain genome via PCR using the primers 5′-GGCGAATTCACCATGTGCCTCTTACATATTTC-3′ and 5′-ACTCAAGCTTTTAAATGTCAAACATCTGGT-3′. The PCR product was digested with EcoRI and Hind III and cloned into the EcoRI-HindIII site of the pcDNA 3.1 (−) vector (Thermo Fisher Scientific). Flag tag sequences (5′-GACTACAAAGACGATGACGACAAG-3′) were inserted at the 5′ end of cloned ORF12 gene using inverse PCR and subsequent self-ligation. The resulting ORF12 expression vector was designated as pcDNA-ORF12 flag. pUC19 vector containing IE promoter region (from +78 to −1807 of IE gene) [9] at Hind III site was generated using GenScript. This plasmid was digested with HindIII and the gel-purified fragment was cloned into the pGL4.10 [luc2] vector (Promega). To check whether the insert was cloned in the proper direction, the cloned plasmid was digested with EcoRV, which exists at the multi-cloning site of the vector and in the cloned sequences, and the length of the fragment was determined using gel electrophoresis. The resultant plasmid was designated as pGL4-IE promoter-Luc.

### 2.8. Luciferase Reporter Assay

Undifferentiated Rn33B-A68B2M cells were seeded in a 6-well plate (Greiner Bio-One) and cultured at 32 °C. Two days after seeding, cells (≈70% confluent) were co-transfected with 0.8 μg each of pcDNA-IE pancRNA, pGL4-IE promoter-Luc, and pcDNA-ORF12 flag using Lipofectamine 3000 (Thermo Fisher Scientific) according to the manufacturer’s instructions. As a negative control, pcDNA3.1 (+) cells were transfected instead of the pcDNA-IE pancRNA. At 24 h after transfection, the cells were lysed with Passive Lysis Buffer (Promega) and assayed for firefly luciferase activity using Luciferase Assay Reagent II (Promega).

To examine whether the Renilla luciferase vector could be used as an internal control reporter vector, three Renilla luciferase vectors with different promoters, pGL4.73[*hRluc*/SV40] vector (Promega), pGL4.74[hRluc/TK] vector (Promega), pGL4.75[hRluc/CMV] vector (Promega), were used; 1.0 μg each of pGL4-IE promoter-Luc and pcDNA-ORF12 flag and 0.1 μg of renilla luciferase vector were transfected and lysed as described above. Renilla luciferase activity was analyzed using the Stop and Glo Reagent (Promega). To evaluate the effects of the ORF12 protein on the expression of Renilla luciferase, cells transfected with vectors, except for pcDNA-ORF12 flag, were assayed.

Normalized firefly luciferase activity was calculated by dividing firefly luciferase activity (RLU) by the amount of firefly plasmid incorporated into cells via transfection. Plasmid DNA was extracted together with cellular DNA from cell pellets in the lysate of reporter assay using Wizard^®^ GenomicDNA Purification Kit (Promega) according to the manufacturer’s instructions. Ten ng of DNA from each sample was used for qPCR with the primers Firefly LuciferaseF and Firefly Luciferase_R [26] (Table 1). Serial 10-fold dilutions of the pGL4-IE promoter-Luc plasmid were used to generate a standard curve. The following real-time PCR program was used: 95 °C for 20 s and 40 cycles of 95 °C for 3 s and 60 °C for 20 s. The RLU divided by the estimated amount of firefly plasmid (ng) in 10 ng of extracted DNA was used as the normalization value.

### 2.9. Construction of IE pancRNA-Expressing Rn33B-A68B2M Cells

The self-inactivating lentiviral vector construct (pCSII-CMV-MCS-IRES2-Bsd), packing construct (pCAG-HIV-gp), and VSV-G and Rev-repression constructs (pCMV-VSV-G-RSV-Rev) were kindly provided by Dr. Miyoshi (RIKEN Bio Resource Center, Ibaraki, Japan). The DNA fragment containing IE pancRNA was digested from pcDNA-IE pancRNA with NheI and XhoI, and the fragment was cloned into the NheI-XhoI site of the pCSII-CMV-MCS-IRES2-Bsd vector. A recombinant lentiviral vector was generated via transient transfection of 293 T cells with a combination of pCAG-HIVgp and pCMV-VSV-G-RSV-Rev. The supernatant containing the lentiviral vector was collected after incubation of the cells at 37 °C for 48 h. Rn33B-A68B2M cells were infected with a lentiviral vector and cultured in the presence of 5 μg/mL blasticidin S (Merck, Darmstadt, Germany). The cells were designated as Rn33B-A68B2M-IE pancRNA cells.

### 2.10. Flow Cytometry

3.0 × 10^5^ undifferentiated Rn33B-A68B2M-IE pancRNA cells were seeded in a 6-well plate (Falcon, Corning, NY, USA) and cultured at 32 °C. Two days after seeding, cells were infected with EHV-1 Ab4-GFP at an MOI of 0.05. At 24 and 48 h p.i., the cells were harvested, dissociated, and analyzed using a BD FACSVerse system (BD Biosciences, Franklin Lakes, NJ, USA), and the data were analyzed using FACSuite (BD Biosciences). Undifferentiated Rn33B-A68B2M cells were used as negative controls.

### 2.11. Statistical Analyses

Statistical analyses were conducted using Microsoft Excel (Microsoft Corporation 2017) and R Statistical Software (version 4.0.3; R Core Team 2020). Student’s *t*-test was used to analyze the differences between two groups, whereas Dunnett’s test was used to analyze the differences among multiple groups. Statistical significance was set at *p* < 0.05.

## 3. Results

### 3.1. Identification of a Novel ncRNA Expressed in the Upstream Region of IE Coding Sequences in EHV-1-Infected Rn33B-A68B2M Cells

**Figure 1:**
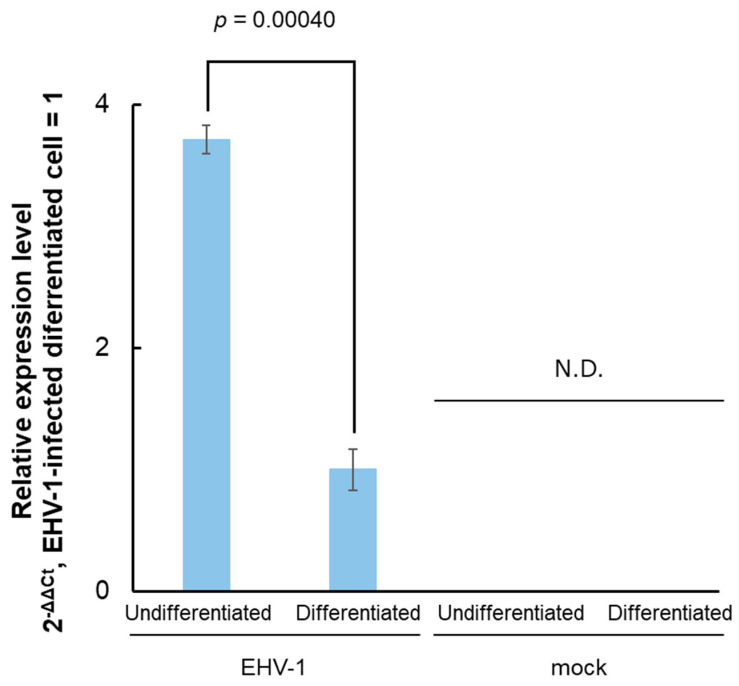
RNA expression levels from the promoter region of the immediate-early (*IE*) gene in equine herpesvirus-1 (EHV-1)-infected Rn33B-A68B2M cells. Undifferentiated and differentiated Rn33B-A68B2M cells were infected with EHV-1 at a multiplicity of infection (MOI) of 5. Total RNA was extracted 24 h post-infection (p.i.). RNA transcribed from the IE promoter region was detected via reverse transcription-quantitative polymerase chain reaction (RT-qPCR). β-actin was used as an internal control. Bar represents the mean with standard deviation from three independent experiments. Student’s *t*-test.

We examined whether the ncRNAs are expressed in the EHV-1 IE promoter region of EHV-1-infected cells. The Rn33B-A68B2M rat neuronal cell line was used to investigate viral gene expression in both undifferentiated and neuronally differentiated EHV-1-susceptible and -unsusceptible cells [22]. RT-qPCR was performed using primer pairs amplifying a part of the IE promoter (nt. 143841–143943) to detect the RNA expression in both EHV-1-infected undifferentiated and differentiated cells (Figure 1). Expression levels were higher in the undifferentiated cells than in the differentiated cells but were undetectable in the uninfected cells.

### 3.2. Determination of the 5′- and 3’-Ends of ncRNA via RACE

**Figure 2:**
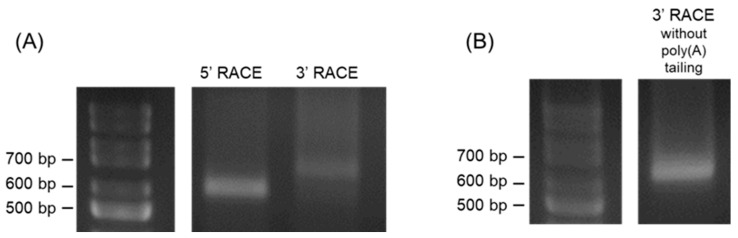
5′- and 3′-rapid amplification of cDNA ends (RACE) of the IE promoter-associated RNA in EHV-1-infected Rn33B-A68B2M cells. Undifferentiated Rn33B-A68B2M cells were infected with EHV-1 at an MOI of 5. Total RNA was extracted at 24 h p.i. (**A**). Gel electrophoresis images of PCR products from the 5′- and 3′-RACE assays of ncRNA in the antisense orientation of the IE promoter. (**B**). Gel electrophoresis images of PCR products from the 3′-RACE assay of ncRNA without the addition of a poly(A) tail.

Next, we performed 5’- and 3’-RACE analyses to determine the full length of IE promoter-associated RNA. We could amplify 5’- and 3’-ends of RNA transcribed in the antisense direction (Figure 2A). Nucleotide sequences of the 5’- and 3’-ends revealed that the antisense RNA was transcribed from the nt region 144287–143290 of the EHV-1 genome, which is equivalent to the position from +344 to –654 in the IE TSS. Moreover, 3’-RACE without poly (A) tail in cDNA synthesis resulted in amplification similar to that of 3’-RACE with poly (A) tail (Figure 2B), suggesting that the antisense RNA has a polyadenylated tail. However, we could not amplify the 5’- and 3’- ends of the RNA transcribed in the sense direction.

**Figure 3:**
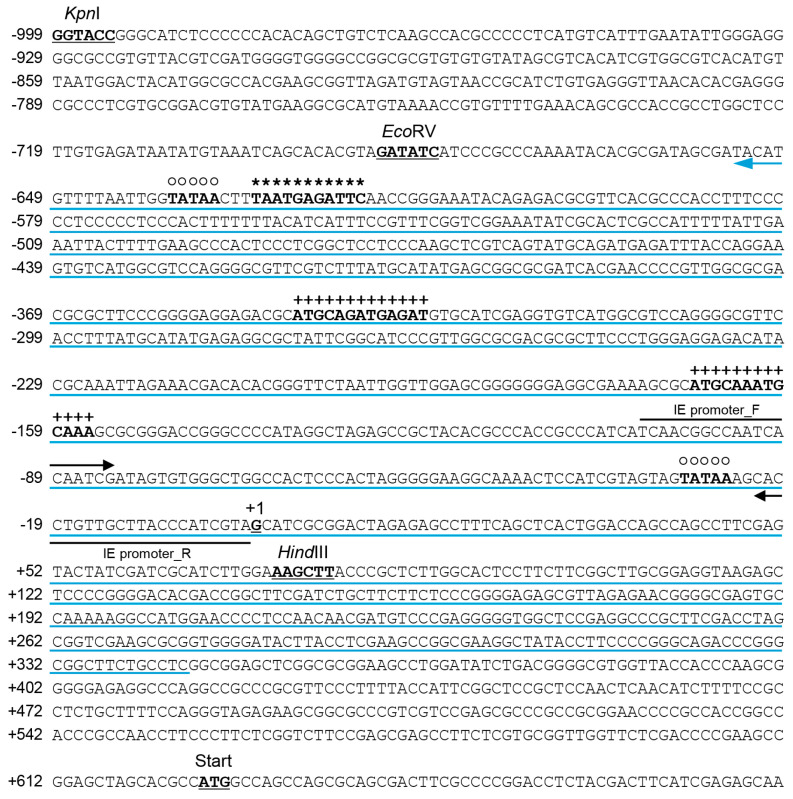
Map of pancRNA transcription in the upstream regulatory region of the EHV-1 *IE* gene. The transcription start site of IE mRNA is designated +1. The position between −999 and +681, which is equivalent to nucleotide region 142945–144624 of the EHV-1 genome (GenBank accession number NC_001491.2), is shown. IE pancRNA is transcribed in the antisense orientation from +344 to −654 as indicated by a blue arrow. Black arrows indicate the annealing sites of primers (EHV-1 IE promoter_F and R) used for initial detection of IE promoter-associated RNA. *Kpn*I-*Hind*III and *Eco*RV-*Hind*III fragments are reported to act as IE promoters in the presence of herpesvirus α-trans-inducing factor (TIF) [8]. A TAATGARATTC motif (*) presented at position −630 to 620 is the binding site for the αTIF–transcription complex [27]. Octamer DNA binding sites (+) at positions −346 to −334 and −168 to −156 are reported to be involved in EHV-1 ORF 12-activated IE gene transcription [9]. Possible TATA boxes (○) are marked at positions −638 and −29. ATG at position +626 is the start codon for IE.

Antisense RNA predicted to be transcribed from the region overlapping the 5′-untranslated and promoter regions (from +344 to −654) of IE was designated as the IE promoter-associated ncRNA (IE pancRNA) (Figure 3).

### 3.3. IE pancRNA Is Expressed in EHV-1-Infected RN33B-A68B2M, RK13, and E. Derm Cells

**Figure 4:**
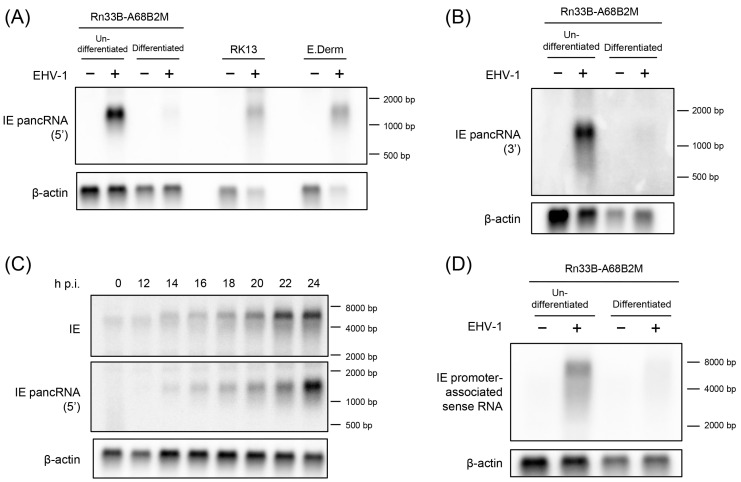
Expression levels of the IE promoter-associated non-coding RNA (pancRNA) in Rn33B-A68B2M, RK13, and E. Derm cells. Cells were infected with EHV-1 at an MOI of 5. Total RNA was extracted from EHV-1- and mock-infected cells at 24 h p.i. (**A**) Northern blotting with digoxigenin (DIG)-labeled RNA probe that hybridizes to the 5′ region of IE pancRNA. (**B**) Northern blotting with DIG-labeled RNA probe that hybridizes to the 3′ region of IE pancRNA. (**C**) Time course of the expression levels of IE pancRNA and *IE* gene in EHV-1-infected undifferentiated RN33B-A68B2M cells. (**D**) Northern blotting with DIG-labeled RNA probe that hybridizes to the sense transcript from the IE promoter region.

The expression and length of IE pancRNA, as expected upon RT-qPCR and RACE analyses, respectively, were validated using northern blot hybridization using Rn33B-A68B2M cells. Undifferentiated and differentiated Rn33B-A68B2M cells were infected with EHV-1 Ab4 at an MOI of 5, and total RNA was extracted at 24 h p.i. Uninfected Rn33B-A68B2M cells were used as the negative control. We prepared two strand-specific probes complementary to the 5′ side and 3′ side of IE pancRNA. The IE pancRNA was detected approximately 1.3 kb in length in EHV-1-infected undifferentiated and differentiated Rn33B-A68B2M cells treated with both probes (Figure 4A, left side, B). Consistent with the qPCR data (Figure 1), the expression levels were higher in undifferentiated cells than in differentiated cells.

Next, we examined whether pancRNA expression preceded or lagged IE gene expression in EHV-1-infected Rn33B-A68B2M cells. Undifferentiated Rn33B-A68B2M cells were infected with EHV-1 at an MOI of 5. RNA was extracted at 0, 12, 14, 16, 18, 20, 22, and 24 h p.i. and hybridized to probes specific for IE pancRNA or IE transcript. Both IE pancRNA and IE transcripts were detected at the same time point, as early as 14 h p.i., using northern blot hybridization (Figure 4C).

To confirm whether IE pancRNA was expressed in other cell lines, northern blot analysis was performed in RK13 and E. Derm cells, which are highly susceptible to EHV-1 infection. RK13 cells were infected with EHV-1 Ab4 at an MOI of 0.5, and RNA was extracted at 12 h p.i. E. Derm cells were infected with EHV-1 at an MOI of 5 and RNA was extracted at 24 h p.i. Uninfected cells were used as negative controls. Similar to Rn33B-A68B2M cells, RK13 and E. Derm cells also showed IE pancRNA expression upon EHV-1 infection (Figure 4A, right side).

We also performed northern hybridization using a probe for the IE promoter-associated sense RNA in Rn33B-A68B2M cells. Weak transcript signals, spanning the IE promoter region approximately 6.7 kb in length, were detected in EHV-1-infected undifferentiated and differentiated Rn33B-A68B2M cells (Figure 4D). This may have been caused by the detection of IE mRNA transcribed upstream of a known transcription initiation site.

### 3.4. IE pancRNA Promotes IE Gene Transcription

**Figure 5:**
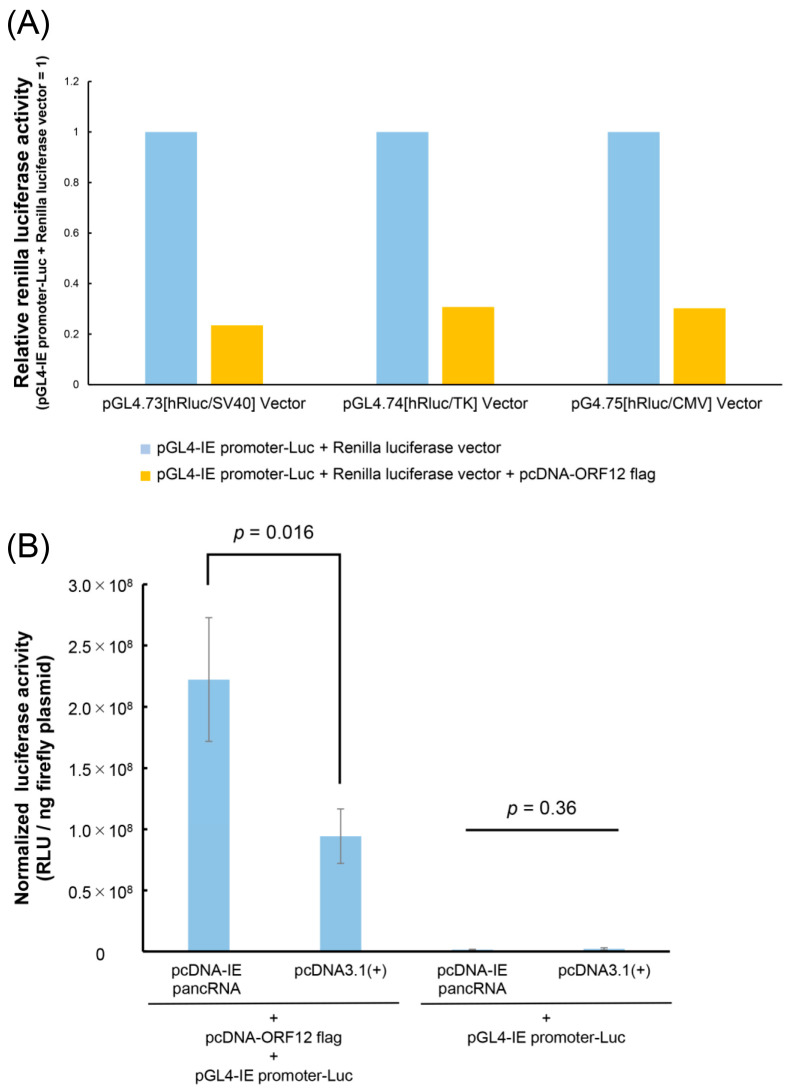
Effect of IE pancRNA on IE promoter activity in undifferentiated Rn33B-A68B2M cells. Undifferentiated Rn-33B-A68B2M cells were co-transfected with luciferase reporter plasmids containing IE promoter (pGL4-IE promoter-Luc), IE pancRNA expression vector (pcDNA-IE pancRNA), and ORF12 expression vector (pcDNA-ORF12 flag). At 24 h p.i., cells were lysed and assayed for luciferase activities. (**A**) Firefly/Renilla dual luciferase assay was performed to verify whether the renilla luciferase vector with Simian virus 40 (pGL4.73[*hRluc*/SV40] vector), thymidine kinase (pGL4.74[*hRluc*/TK] vector), or cytomegalovirus (pGL4.75[*hRluc*/CMV] vector) promoters can be used as an internal control. All samples were analyzed in singlicate. (**B**) Firefly luciferase assay was used to examine the role of IE pancRNA in *IE* gene transcription. Normalized firefly luciferase activity was calculated by dividing the firefly luciferase activity (RLU) by the firefly plasmid amount (ng) incorporated into cells via transfection. Bar represents the mean with standard deviation from three independent experiments. Student’s *t*-test.

To investigate the role of IE pancRNAs in IE gene transcription, a luciferase reporter assay was performed. EHV-1 ORF12 protein was previously reported as a transactivator of *IE* gene expression [28,29,30]. Control reporter vectors containing constitutive promoters (SV40, HSV-TK, or CMV) were not used to normalize Luc activity as EHV-1 ORF12 significantly activated these promoters (Figure 5A). Therefore, in the following experiments, normalization was performed by dividing firefly luciferase activity by the amount of firefly plasmid incorporated by cells via transfection. In the presence of ORF12, cells transfected with the IE pancRNA expression vector exhibited higher RLU than those transfected with the empty control vector (Figure 5B). In the absence of ORF12, RLU was low, regardless of the expression of IE pancRNA. These results suggest that IE pancRNA promotes the transcription of *IE* via ORF12.

We next examined the possibility of the involvement of IE pancRNA-derived protein in the promotion of IE gene transcription. DNA sequence from which pancRNA was transcribed was analyzed using ORF finder (https://www.ncbi.nlm.nih.gov/orffinder/, accessed on 30 May 2024), and five potential ORFs were found (Table S1). To inhibit the possible protein translation from IE pancRNA, we introduced a stop codon near the 5′ end of each ORF or substituted the start codon with another amino acid by replacing one or two nucleotides with other nucleotides through inverse PCR using pcDNA-IE pancRNA and primers with nucleotide substitutions (Figure S1). We then transfected the four mutant pancRNA expression vectors into Rn33B-A68B2M cells and evaluated their effect on IE promoter activity (Figure S2). Similar to the wild-type pancRNA expression vector (pcDNA-IE pancRNA), cells transfected with mutant pancRNA expression vectors showed higher RLU than those transfected with the empty control vector, suggesting that even if proteins derived from these ORFs exist, they are not the primary cause of increased transcription of IE genes by pancRNA.

### 3.5. IE pancRNA Promotes EHV-1 Infection in Rn33B-A68B2M Cells

**Figure 6:**
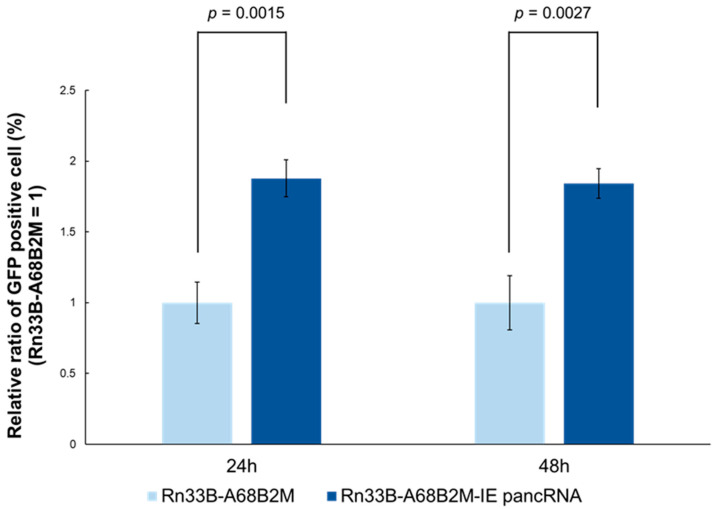
Effect of IE pancRNA expression on EHV-1 infection in undifferentiated Rn33B-A68B2M cells. Undifferentiated Rn33B-A68B2M cells expressing IE pancRNA (Rn33B-A68B2M-IE pancRNA) and undifferentiated Rn33B-A68B2M cells were infected with Ab4-green fluorescent protein (GFP) at an MOI of 0.05. At 24 and 48 h p.i., the cells were harvested, dissociated, and the number of GFP-positive cells was quantified via flow cytometry. Bar represents the mean with standard deviation from three independent experiments. Student’s *t*-test.

Finally, the effect of IE pancRNA on EHV-1 infection was examined using Rn33B-A68B2M cells stably expressing pancRNA (Rn33B-A68B2M-IE pancRNA). At 24 and 48 h p.i. with GFP-expressing EHV-1, the number of GFP-positive cells in the Rn33B-A68B2M-IE pancRNA group was higher than that in the Rn33B-A68B2M group (Figure 6). This suggests that IE pancRNA promotes EHV-1 infection in Rn33B-A68B2M cells.

## 4. Discussions

In this study, we identified a novel ncRNA, named IE pancRNA, transcribed from the EHV-1 IE 5′-untranslated and promoter regions in EHV-1-infected cells. IE pancRNA promoted the transcription of the *IE* gene and promoted EHV-1 infection in Rn33B-A68B2M. The involvement of ncRNAs in EHV-1 infections remains largely unknown. To the best of our knowledge, this is the first report of a virus-derived lncRNA regulating EHV-1 expression.

PancRNAs are lncRNAs transcribed from bidirectional promoters regulating the expression of protein-coding genes [19]. Recent studies revealed the mechanisms underlying the regulation of gene expression by pancRNAs. pancRNA expression in the promoter region of vimentin induces sequence-specific DNA demethylation in the promoter region to promote transcription [21]. Myoparr, which is expressed in the promoter region of myogenin, is presumed to promote myogenin expression by regulating the association between the transcriptional coactivator and histone acetyltransferase [31]. Here, our study revealed IE pancRNA as a functional pancRNA regulating paired gene transcription. However, the underlying mechanisms, especially those responsible for enhanced transactivation by ORF12, remain unknown. RACE analysis revealed that the 5′-end of IE pancRNA is located downstream of the IE TSS. Although the majority of pancRNAs are transcribed upstream of the TSS of a paired gene, a functional pancRNA transcribed from 1161 bp downstream of the TSS of *Nefl* in rat PC12 cells causes DNA demethylation in *Nefl* upstream region [21].

We identified IE pancRNA as a 998-nucleotide transcript (nt. 144287–143290) using RACE. However, an approximately 1.3-kb band was detected in EHV-1-infected cells via northern blotting for IE pancRNA. This discrepancy may be due to the addition of a long (approximately 200–300 bp) poly(A) tail [32]. LncRNAs can have a 5′-cap structure and/or be spliced similar to mRNAs, warranting further analyses on the post-transcriptional modification of IE pancRNA [33].

Here, we demonstrated that IE pancRNA was expressed in the EHV-1-infected RK13 rabbit-kidney-derived, E. Derm equine dermal, and Rn33B-A68B2M rat neuronal cell lines. Our findings suggest that IE pancRNA is expressed in various cell types and regulates EHV-1 gene expression during EHV-1 lytic infection.

In conclusion, we identified a pancRNA transcribed from the IE promoter region that enhanced EHV-1 infection. Moreover, our findings suggest the potential involvement of IE pancRNA, which upregulated IE gene transcription, in EHV-1 infection mechanisms in horses. However, further studies are necessary to validate these findings.

## Figures and Tables

**Table 1:** Nucleotide sequences of primers used for reverse transcription-quantitative polymerase chain reaction (RT-qPCR) and rapid amplification of cDNA ends (RACE).

Primer	Sequence (5′–3′)	
EHV-1 IE promoter_F	TCAACGGCCAATCACAATCG	(20 bp)
EHV-1 IE promoter_R	TACGATGGGTAAGCAACAGGTG	(22 bp)
Rat_β-actin_F	AAGTCCCTCACCCTCCCAAAAG	(22 bp)
Rat_β-actin_R	AAGCAATGCTGTCACCTTCCC	(21 bp)
5′ RACE Antisense GSP	GATTACGCCAAGCTTCGACACACGGGTTCTAATTGGTTGGAG	(42 bp)
3′ RACE Antisense GSP	GATTACGCCAAGCTTCTACGATGGAGTTTTGCCTTCCCCCTA	(42 bp)
5′ RACE Sense GSP-1	GATTACGCCAAGCTTTACGATGGAGTTTTGCCTTCCCCCTAGT	(43 bp)
5′ RACE Sense GSP-2	GATTACGCCAAGCTTCTCCAACCAATTAGAACCCGTGTGTCG	(42 bp)
3′ RACE Sense GSP-1	GATTACGCCAAGCTTACGACACACGGGTTCTAATTGGTTGGAG	(43 bp)
3′ RACE Sense GSP-2	GATTACGCCAAGCTTCGCTTCCCTGGGAGGAGACATACGCAAA	(43 bp)
3′ RACE Sense GSP-3	GATTACGCCAAGCTTCACTAGGGGGAAGGCAAAACTCCATCG	(42 bp)
Firefly Luciferase_F	CACCGTCGTATTCGTGAGCA	(20 bp)
Firefly Luciferase_R	AGTCGTACTCGTTGAAGCCG	(20 bp)

Abbreviation: bp, base pair.

## Data Availability

All data generated or analyzed in this study are included in the published article.

## Supplementary Materials

The following supporting information can be downloaded at: https://www.mdpi.com/article/10.3390/v16081195/s1, Table S1: Possible open reading frames in IE pancRNA; Figure S1: Construction of IE-pancRNA expression vectors with nonsense or start-loss mutations in predicted ORFs; Figure S2: Effect of nonsense or start-less mutation of potential ORFs in IE pancRNA.
