# Supplementary material for: Identification of the Promoter Antisense Transcript Enhancing the Transcription of the Equine Herpesvirus-1 Immediate-Early Gene
Source: Viruses. 2024 Jul 25;16(8):1195. doi: 10.3390/v16081195 (PMC11360796; doi:10.3390/v16081195)
Supplement: Supplementary file 1 [file viruses-16-01195-s001.zip › viruses-3031879-supplementary.pdf]

Table S1. Possible open reading frames in IE pancRNA

	Frame[a]	Start[b]	Stop[b]	Length (nt | aa)
ORF 1	1	142	291	150	49
ORF 2	1	349	447	99	32
ORF 3	2	341	436	96	31
ORF 4	3	279	317	39	12
ORF 5	3	447	734	288	95

[a]Number of the reading frames in 5' to 3' direction of IE pancRNA

[b]Nt number of IE pancRNA

Abbreviation: nt, nuleotide; aa, amino acid

```
ORF 1 (MAFLALAPFSNALPGRRSRSKPVVSPGSSYLRKPKKECQERVSFPRCDR)
nt 139
     M   A   F   L   A   L   A   P   F   S
TCC ATG GCC TTT TTG GCA CTC GCC CCG TTC TCT … : pcDNA-IE pancRNA
TCC ATG GCC TTT TAG GCA CTC GCC CCG TTC TCT … : pcDNA-IE panc T152A
     M   A   F   *

ORF 2 (MGKQQVLYTTTMEFCLPPSGSGQPTLSIVIGR)
nt 346
     M   G   K   Q   Q   V   L   Y   T   T
ACG ATG GGT AAG CAA CAG GTG CTT TAT ACT ACT … : pcDNA-IE pancRNA
ACG ATA GGT AAG CAA CAG GTG CTT TAT ACT ACT … : pcDNA-IE panc G351A
     I

ORF 3 (MLRWVSNRCFILLRWSFAFPLVGVASPHYRL)
nt 338
     M   L   R   W   V   S   N   R   C   F
GCG ATG CTA CGA TGG GTA AGC AAC AGG TGC TTT … : pcDNA-IE pancRNA
GCG ATG CTA CGA TAG GTA AGC AAC AGG TGC TTT … : pcDNA-IE panc G351A
     M   L   R   *

ORF 4 (MRSIVLEGWLVQ)
nt 276
     M   R   S   I   V   L   E   G   W   L
AAG ATG CGA TCG ATA GTC CTC GAA GGC TGG CTG … : pcDNA-IE pancRNA
AAG ATG CGA TCG TAA GTC CTC GAA GGC TGG CTG … : pcDNA-IE panc A288T/T289A
     M   R   S   *

ORF 5
(MMGGGRVAALAYGARSRALHLHALFASPPLQPIRTRVSFLICVCLLPGKRVAPTGCRIAPLICIKVNAPGRHDTSMH
ISSACVSSPGSASRQRGS)
nt 444
     M   M   G   G   G   R   V   A   A   L   A   Y   G
TTG ATG ATG GGC GGT GGG CGT GTA GCG GCT CTA GCC TAT GGG  … : pcDNA-IE pancRNA
TTG ATG ATG GGC GGT GGG CGT GTA GCG GCT CTA GCC TAA GGG  … : pcDNA-IE panc
     M   M   G   G   G   R   V   A   A   L   A   *              T482A
```

Figure S1. Construction of IE-pancRNA expression vectors with nonsense or start-loss mutations in predicted ORFs.

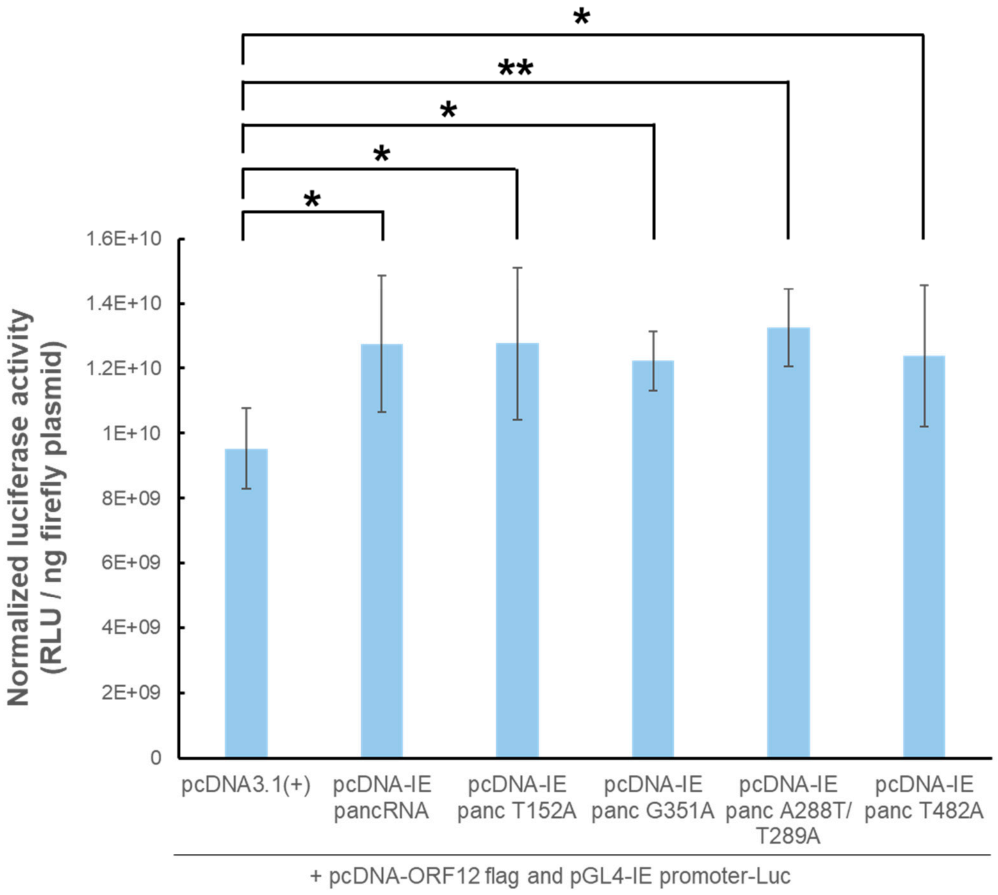

Figure S2. Effect of nonsense or start-less mutation of potential ORFs in IE pancRNA. Undifferentiated Rn33B-A68B2M cells were co-transfected with luciferase reporter plasmids containing IE promoter (pGL4-IE promoter-Luc), wild type IE pancRNA expression vector (pcDNA-IE pancRNA) or mutant IE pancRNA expression vectors (pcDNA-IE panc T152A, pcDNA-IE panc G351A, pcDNA-IE panc A288T/T289A, and pcDNA-IE panc T482A), and ORF12 expression vector (pcDNA-ORF12 flag). At 24 h p.i., cells were lysed and assayed for luciferase activities. Normalized firefly luciferase activity was calculated by dividing the firefly luciferase activity (RLU) by the firefly plasmid amount (ng) incorporated into cells via transfection. Each bar represents the mean of six independent samples. Error bars show standard deviations. **$P < 0.01$, *$P < 0.05$. Dunnett's test.
